# Clinical Efficacy of Trimetazidine and Holistic Management in the Treatment of Coronary Heart Disease

**Published:** 2018-06

**Authors:** Li QU, Hua JIANG

**Affiliations:** The Second Affiliated Hospital of Dalian Medical University, Dalian, Liaoning 116023, P.R. China

**Keywords:** Coronary heart disease, Trimetazidine, Clinical efficacy, Nursing experience

## Abstract

**Background:**

To investigate the clinical efficacy of trimetazidine and holistic management in the treatment of coronary heart disease.

**Methods:**

A total of 128 cases of patients with coronary heart disease were admitted in The Second Affiliated Hospital of Dalian Medical University from October 2014 to June 2017. These patients were divided into control group and experimental group, with 64 patients each. The patients in the control group underwent conventional treatment. On this basis, the patients in the experimental group were treated with trimetazidine. Both groups underwent holistic management. The clinical conditions, echocardiography indexes, life quality, and mental states of patients were compared between the two groups.

**Results:**

The total efficient rate of patients in the control group was significantly lower than that of the experimental group, and the difference was statistically significant (*P*<0.05). After treatment, the left ventricular ejection fraction of patients in the experimental group was higher than that of the control group; the left ventricular end diastolic diameter and left ventricular posterior wall thickness of patients in the experimental group were lower than those of the control group, and the differences were statistically significant (*P*<0.05). The physiological health score, mental health score, Hamilton Depression Scale and Hamilton Anxiety Rating Scale of patients were significantly decreased compared with the control group, and the differences were statistically significant (*P*<0.05).

**Conclusion:**

The efficacy of trimetazidine in the treatment of coronary heart disease is definite. The assisting holistic management can significantly improve the mental status and life quality of patients by enhancing the cardiac function, which has clinical reference and promotion values.

## Introduction

Coronary heart disease is a common clinical disease. The occurrence of this condition is higher in the elderly population and shows a significant rejuvenated trend (1, 2). This disease has more complications and comorbidities and has higher mortality, which seriously threatens the life safety and physical and mental health of vast majority of patients (3). Trimetazidine is a piperazine derivative, which can significantly reduce the myocardial oxygen consumption, optimize the myocardial energy metabolism, and maintain the myocardial oxygen supply balance (4).

We investigated the clinical efficacy of trimetazidine and holistic nursing method in the treatment of coronary heart disease. On this basis, the assisting holistic management intervention can ensure the successful development of therapy and further improve the mental status and life quality of patients. A total of 128 cases of patients with coronary heart disease admitted in our hospital were involved in this paper.

## Methods

A total of 128 cases of patients with coronary heart disease admitted in The Second Affiliated Hospital of Dalian Medical University from October 2014 to June 2017 were enrolled. All patients were in accordance with the diagnostic criteria of coronary heart disease formulated by WHO. The patients with atrial flutter, atrial fibrillation, liver and kidney dysfunction, and hypertrophic heart disease were not included. Following the method of random sampling, the patients were divided into control group and experimental group, with 64 patients each. Thirty-five cases of males and 29 cases of females were in the control group. In the control group, the minimum age of patients was 46 years, the maximum age was 85 years old, and the average age was 67.4 ± 4.5 years.

The duration was 1 to 9 years, and the average duration was 4.7±1.3 years. Moreover, 38 cases of males and 28 cases of females were in the experimental group. In the experimental group, the minimum age of patients was 47 years, the maximum age was 86 years, and the average age was 68.2±4.3 years. The course was 1 to 10 years, and the average duration was 4.8±1.4 years. The baseline data of patients were compared between the two groups, and no statistically significant difference was observed between the two groups (*P* > 0.05).

### Therapy

The patients in the control group underwent conventional therapy; nitrate was used to dilate the coronary artery, and the channel blocker was used to significantly increase the myocardial contractility. Meanwhile, ACEI, β-receptor blocker, and low-dose aspirin were administered simultaneously. On this basis, the patients in the experimental group were treated with 20 mg of trimetazidine, tid (national medicine quasi word H20055465, Servier (TIAN JIN) Pharmaceutical Co., Ltd., drug specifications: chemical drugs, 20 mg).

The study was approved by the Ethics Committee of The Second Affiliated Hospital of Dalian Medical University and informed consents were signed by the patients and/or guardians.

### Holistic management

Symptomatic nursing intervention: The vital signs of patients were monitored. If the emotional fluctuation is bigger, the doctor’s advice should be strictly followed, and the sedative drugs should be adopted. Meanwhile, the language pacifying and counseling should be conducted. The patients’ myocardial oxygen consumption should be reduced as far as possible, and the oxygen demand should be met through persistent oxygen uptake.

Psychological intervention: The patients with coronary heart disease are often accompanied by severe mood changes and poor mental status. The nursing staff should introduce the disease-associated knowledge in detail for the patients and their families. This orientation is for the following objectives: to improve the correct cognition and understanding of coronary heart disease, to support and encourage the patients, to teach them reasonable emotional disclosure, to patiently listen to the patients’ thought, to answer the related questions and problems raised by patients in time, and to strengthen the psychological support and intervention of patients. The experience exchange of patients can be increased, so as to persuade and encourage each other. The previous good therapy case should be introduced to help patients establish determination against the disease and to improve the coordination ability and treatment compliance.

Rehabilitation intervention: According to the specific condition of patients, the individualized rehabilitation archives were formulated in detail; every activity of patients were recorded in detail. The activity time and frequency were reasonably adjusted and combined with the actual situation of patients. The elderly patients’ understanding ability is poor, so the medical staff should demonstrate in person and explain at the same time. This act is for the patients to be aware of the importance of rehabilitation nursing and actively cooperate with the completion of the treatment and medical work.

Strengthen the follow-up visit: The in-charge staff should formulate the follow-up archives for the discharged patients. Furthermore, the nurses should explore and understand the specific conditions of patients through the family follow-up visit. The patients should visit the hospital regularly for ECG examination after being discharged. The health education about the disease knowledge and nursing should be performed. The patients should be debriefed to maintain the emotional stability in daily life and to strictly control the dosage and usage of medications. Moreover, the arbitrarily drug increase or reduce should be prohibited. If the patients feel abnormality or physical discomfort, then the review should be conducted as soon as possible.

### Observation index

Echocardiography: LVEF (left ventricular ejection fraction), LVD (left ventricular end diastolic diameter), and LVWP (left ventricular posterior wall thickness).

Quality of life: The patients’ life quality was assessed using the MCS (mental health score) and PCS (physiological health score). MCS mainly includes emotional function, social function, vitality, and mental health. PCS mainly covers body pain, physiological function, general health, and physiological role. The scores are directly proportional to the health status and life quality of patients.

Mental state: The patients’ depressive state was assessed using the HAMD (Hamilton Depression Scale). The patients’ neurological condition was assessed using the SSS (stroke neurological impairment scale). The higher the score, the more severe the depression and neurological deficit.

### Efficacy evaluation criteria

Excellent: The clinical symptoms and signs of patients basically disappeared after the nursing treatment. The improvement of cardiac function was greater than level 2 or normal. Effective: The clinical symptoms and signs of patients were relieved after nursing treatment. The improvement of cardiac function was at level 1. Invalid: The clinical symptoms and signs, as well as cardiac function of patients, did not change or was not aggravated after nursing treatment compared with prior condition. The total efficacy rate was the case percentage of excellent and effective rates.

### Statistical processing methods

The data were analyzed using the statistical software SPSS 22.0 (Chicago, IL, USA). The echocardiography indexes, mental states, and life qualities of patients were expressed using (x̄±*s*) and compared using t-test. The total efficacy rate was expressed with the percentage (%) and compared with *χ*^2^ test. P < 0.05 indicates statistically significant difference between the two groups.

## Results

### Comparison of therapeutic effects between the two groups

The total efficacy rate of patients in the experimental group was superior to that of the control group, and a statistically significant difference was observed between the two groups (*P* < 0.05) (Table 1).

**Table 1:** Comparison of therapeutic effects between experimental and control groups [n (%)]

***Group***	***N***	***Excellent***	***Effective***	***Invalid***	***Total efficacy rate***
Control	64	32 (50.0)	22 (34.4)	10 (15.6)	54 (84.4)
Experimental	64	36 (56.3)	26 (40.6)	2 (3.1)	62 (96.9)
*χ*^2^					5.8851
*P*					0.0152

### Comparison of echocardiography indexes between the two groups

After nursing treatment, the LVD and LVWP of patients in the experimental group were lower than those of the control group. The LVEF of patients was higher than that of the control group, and the differences were statistically significant between the two groups (*P* < 0.05) (Table 2).

**Table 2:** Comparison of echocardiography indexes between experimental and control groups (x̄±*s*)

***Group***	***N***	***LVEF (%)***	***LVWP (mm)***	***LVD (mm)***
Control	64	32.84±6.25	16.47±5.77	67.33±6.42
Experimental	64	37.45±5.66	13.62±5.34	64.52±5.73
*t*		4.3738	2.9000	2.6123
*P*		0.0000	0.0044	0.0101

### Comparison of mental state changes between the two groups

The HAMD and SSS scores of patients in the experimental group were lower than those in the control group, and the differences were statistically significant between the two groups (*P* < 0.05) (Table 3).

**Table 3:** Comparison of mental state changes between experimental and control groups (x̄±*s*)

***Group***	***N***	***HAMD score***	***SSS score***
Control	64	29.32±4.48	24.32±5.25
Experimental	64	10.48±3.15	10.39±4.68
*t*		27.5208	15.8450
*P*		0.0000	0.0000

### Comparison of life qualities between the two groups

The PCS and MCS scores of patients in the control group were lower than those of the experimental group, and the differences were statistically significant between the two groups (*P* < 0.05) (Table 4).

**Table 4:** Comparison of life qualities between experimental and control groups (x̄±*s*)

***Group***	***N***	***PCS score***	***MCS score***
Control	64	183.53±49.54	188.89±55.54
Experimental	64	236.14±53.66	243.13±63.73
*t*		5.7629	5.1330
*P*		0.0000	0.0000

## Discussion

Coronary heart disease is one of the most common cardiovascular diseases in clinical practice. This condition has high incidence cases, long duration, and poor prognosis (5). A regular and accurate early medication is the key to improve the disease condition and enhance the life quality of the patient. Trimetazidine is a commonly used drug in the treatment of coronary heart disease, which can optimize the myocardial energy metabolism transfer of patients with unstable angina pectoris from fatty acid oxidation to glucose oxidation and can potently inhibit β oxidation of free fatty acids (6, 7). Moreover, this medication can inhibit the myocardial uptake of fatty acids, significantly decrease the myocardial oxygen consumption, effectively protect the myocardial cells under hypoxia or ischemia, and significantly reduce the damage of ischemia. Simultaneously, trimetazidine can significantly improve the myocardial contractility, reduce the excessive myocardial cell injury, promote the collateral circulation, increase the myocardial blood supply, and significantly improve the hypercoagulable state of ischemic site, which will not affect the blood pressure and heart rate (8). The efficacy is precise.

In recent years, with the continuous improvement of the medical care concept and level, coupled with the establishment and development of biological-social-psychological medical model, the targeted nursing plan is formulated according to the specific conditions of patients (9). Through the targeted medical intervention, the medical problems can be identified as soon as possible, which can be timely dealt with and solved. The holistic management is a new mode arising under this kind of background (10). This comprehensive method, with the medical intervention as basic conditions, is an important link to improve the clinical efficacy. The holistic management experience of patients with coronary heart disease further deepens humanistic care connotation, which can fully embody the people-oriented medical service concept, can make the patients feel more cared for by the management of the medical staff, and can improve the patients’ mental status (11). The rehabilitation training should be performed as soon as possible to accelerate the recovery speed. The patients with coronary heart disease were treated with trimetazidine combined with holistic management, which can play twice of the efficacy and further improve the quality of medical care.

## Conclusion

Trimetazidine is effective in the treatment of coronary heart disease, and the assisting holistic medical care can significantly improve the mental status and life quality of patients and enhance their cardiac function, which has clinical reference and promotion value.

## Ethical considerations

The author completely observed and conformed to all ethical guidelines regarding plagiarism, informed consent, misconduct, data fabrication and/or falsification, double publication and/or submission, and redundancy
